# German and Italian Users of Web-Accessed Genetic Data: Attitudes on Personal Utility and Personal Sharing Preferences. Results of a Comparative Survey (n=192)

**DOI:** 10.3389/fgene.2020.00102

**Published:** 2020-03-18

**Authors:** Sabine Wöhlke, Manuel Schaper, Serena Oliveri, Ilaria Cutica, Francesca Spinella, Gabriella Pravettoni, Daniela Steinberger, Silke Schicktanz

**Affiliations:** ^1^Department of Medical Ethics and History of Medicine, University Medical Center Göttingen, Göttingen, Germany; ^2^Department of Oncology and Hematology Oncology, Faculty of Medicine and Surgery, University of Milan, Milan, Italy; ^3^Applied Research Division for Cognitive and Psychological Science, European Institute of Oncology, Milan, Italy; ^4^Laboratory GENOMA, Rome, Italy; ^5^bio.logis Genetic Information Management GmbH, Frankfurt, Germany

**Keywords:** genomics, health information, attitudes, experiences, survey, lay people, utility

## Abstract

Genetic information is increasingly provided outside of the traditional clinical setting, allowing users to access it directly *via* specialized online platforms. This development is possibly resulting in changing ethical and social challenges for users of predictive genetic tests. Little is known about the attitudes and experiences of users of web-accessed genetic information. This survey analyzes data from two European countries with regard to the utility of genetic information, the users’ ways of making use of and dealing with information, and their sharing behavior. Particular focus is given to ethical and social questions regarding the motivation to share personal genetic results with others. Social factors tested for are national background, gender, and marital, parental, and educational status. This study will contribute to public discourse and offer ethical recommendations. The study will also serve to validate the developed questionnaire for use in population representative surveys.

## Introduction

Lay people are increasingly able to access digitized data regarding their personal health, ranging from information provided by self-tracking and fitness apps to electronic patient records (Lupton, 2014; Rexhepi et al., 2018). Within this trend, genetic information has become widely available, presenting lay people in the role of patients and consumers of health services with a variety of implications and possibilities regarding application, utility and information sharing. Research has shown that the public’s interest in genetic information is high (Townsend et al., 2012), and there are different plausible reasons for that interest: Genetic tests can confirm or rule out genetic traits or a suspected genetic condition, or they can help to determine a person’s chance of developing or passing on a genetic disorder, and in some cases they provide relevant information that can be used to the patient’s benefit (Burke, 2014). In such cases, genetic testing has “clinical utility”. Recently, scholars have discussed the possibility that genetic testing also has “personal utility”, e.g., that it plays a role in shaping individual understandings of disease or personal identities of their carriers (Bunnik et al., 2015; Kohler et al., 2017a; Kohler et al., 2017b; Urban and Schweda, 2018). However, lay understandings of genetic information and its implications diverge from those of experts, and may be shaped by specific life situations, such as experience of disease, personal attitudes and beliefs, and psycho-social circumstances (Oliveri et al., 2015; Oliveri et al., 2016a; Oliveri and Pravettoni, 2018; Oliveri et al., 2018), as well as by cultural background (Raz and Schicktanz, 2016). Lay people’s perceptions are important because they affect both their interest in undergoing genetic testing as well as their interpretations of test results.

Some institutions offering genetic testing provide direct access to own genetic test results *via* specialized online-platforms. This article focuses on users of such direct access to personal genetic information (i.e., lay people in regard to understanding genetic information) and their specific attitudes and behaviors regarding information sharing and the exercise of responsibility within families (e.g., decisions regarding whether or not to inform relatives about their genetic risks or regarding reproductive behavior) (Welch and Burke, 1998; Anderson and Wasson, 2015; Baars et al., 2016). This perspective is relevant because lay people often consider the decision to undergo genetic testing to be an individual choice rather than a socially embedded decision (Corpas, 2012; Schaper et al., 2018). Receiving genetic risk information can potentially cause psychological harm because some conditions are currently untreatable and being affected may lead to stigmatization and discrimination (Slaughter, 2006; Kollek and Lemke, 2008; Ross et al., 2015). Furthermore, ethical conflicts may arise when the needs of the client/patient do not accord with those of other family members or society at large, and genetic counselors are increasingly faced with conflicting obligations, e.g. when there is critical information available that applies to multiple persons with different information preferences (Muthuswamy, 2011). While a moral duty may exist to share genetic information in order to prevent harm to others, the nature of a specific condition and the predicted harm associated with it need to be considered as well (D’Agincourt-Canning, 2001; Parens and Appelbaum, 2019). However, privacy and confidentiality are central issues in genetic testing and making use of and dealing with genetic information, and while there is consensus that individuals are entitled to knowing about existing genetic information, a right not to know has become the central moral norm, especially regarding genetic risk of contracting a disease (Chadwick et al., 2014; Domaradzki, 2015; Lupton and Michael, 2017).

A German study showed that lay people perceive risk information as highly normatively charged, and often as an emotionally significant threat (Wöhlke et al., 2019). It would therefore seem to be necessary to provide lay people with a deeper understanding of risk information and of the limitations of genetic knowledge with respect to one’s own health responsibility (Wöhlke and Perry, 2019). Similar results were found for lay people in Italy, who perceived genetic testing to be very helpful for disease prevention but were simultaneously afraid that a positive result, the detection of a genetic variant, could affect their life planning and leave them without the ability to act to address the risk (Oliveri et al., 2016a). Across Europe, the regulation of genetic testing is focused on the prevention of harm to the individual—therefore, public opinion should be taken into account in the creation of policy and legislation regarding the communication of genetic risk (Oliveri et al., 2016b).

Questions regarding the implications of personal access to genetic information are becoming increasingly important in the eHealth era, where health information is becoming more accessible to lay people in the role of patients and consumers as well as to various other actors in the healthcare sector. Currently, there are significant differences between countries in terms of the political will to implement eHealth, available infrastructure, and actual use of these possibilities. Here, Germany and Italy offer contrasting examples within Europe, with Germany being less advanced than Italy in eHealth implementation efforts (Poss-Doering et al., 2018; Thiel et al., 2019). Italian eHealth initiatives have mainly been in the areas of improving access to health services and availability of patients’ clinical histories, innovating primary care, and redesigning the healthcare services network through Telemedicine (Domenichiello, 2015). For this study, we conducted a survey of Germans and Italians who have access to their personal genetic information in order to gain a deeper insight into the practical and ethical questions associated with accessing and sharing such information.

Sharing of health information for more efficiency in health care and research is a central notion in the eHealth paradigm. Privacy and confidentiality are therefore important issues in relation to personal data that are acknowledged by political decision makers in both Germany and Italy (Thiel et al., 2019). The present notion of sovereignty over one’s own genetic information touches a number of ethical aspects related to both the self-determination and the privacy of patients. However, it is unclear how the autonomy and right to know of individuals can be reconciled with the self-determination and right not to know of their family members. The holder of genetic information has a special responsibility because of its relevance for other biologically related persons (Leefmann et al., 2017). With the introduction of the General Data Protection Regulation (GDPR), a uniform legal requirement for the handling of personal data was adopted in the European Union, aimed at guaranteeing data security and data sovereignty. However, there is great variety in how genetic testing is legally defined and regulated internationally (Borry et al., 2012; Soini, 2012; Varga et al., 2012). In both Germany and Italy, genetic testing for medical purposes is subject to legislation that requires specialized physicians and the provision of genetic counseling (Kalokairinou et al., 2018).

## Aim

Given the topic outlined above, the overall aim of this study was to gather information about the personal experiences and moral and social attitudes of lay people as well as their ways of making use of and coping with genetic information and examine the similarities and differences between German and Italian users (lay people) of direct access to personal genetic information, and the way these similarities and differences are related to age, sex, and social and educational background.

## Methods

In 2018, we conducted an online survey of persons with direct access to their own genetic information, provided *via* centers for human genetics in Germany and Italy.

The survey consisted of 13 questions in three thematic blocks (see **Supplementary 1**):

- Experience with genetic testing: questions concerning the level of understanding of own genetic test results and perceived controllability of their implications for health.
- Personal opinion on genetic testing in general: questions mainly concerning the utility of genetic testing, who should undergo genetic testing, the right to know or not to know, and regulation.
- Making use of and dealing with test results: questions mainly concerning preferences and reasons in sharing genetic own genetic information.

Further, the survey included a set of sociodemographic questions to contextualize the answers. The survey was initially developed in German by the Göttingen research group. Its content was developed based on the research question and tailored to the target population based on previous experience in studying lay perspectives on genetic testing with qualitative methodology. The survey was adapted and improved in close cooperation with the heads of GenomaLab and bio.logis Zentrum für Humangenetik (ZfH) to meet the practicalities of conducting the survey based on those institutions’ technical infrastructure. Critical feedback from all co-authors was included at an early stage of development. The survey was successfully tested with academic staff of the German and Italian research groups’ affiliation before application in the study. The survey was translated into English by the Göttingen research group, and thence from English to Italian for application by the research groups in Italy. The Italian translation was checked by translating it back into English.

In order to participate in the survey, participants had to read and acknowledge the study information telling them that by proceeding to the questionnaire and submitting it they gave consent to participate.

## Recruitment

We recruited participants who had undergone genetic testing and had online access to their personal genetic information. In the following sections we provide links to a sample account for each website.

### German Sample

Participants were recruited *via* bio.logis (ZfH) in Frankfurt (Main). bio.logis (ZfH) is a clinical institute for pre- and postnatal genetic diagnostics and counselling which provides a web-portal designed to give patients direct access to selected categories of genetic information. Online access to genetic information is offered only for selected categories, such as pharmacogenetics, carrier status for recessively inherited diseases, and preventive targets. Non-treatable conditions or those whose diagnosis would lead to relatively invasive treatments, such as pronounced surgical or chemotherapeutical interventions, were excluded. Patients may log in to their personal account and see the current status of genetic analyses and results as well as news and updates provided by bio.logis (ZfH).^1^ The User ID for access to the portal is provided directly to patients and to their doctors, who in the majority of cases were responsible for the referrals of patient’s samples. For the purpose of recruitment users were contacted *via* an internal e-mail system of the bio.logis (ZfH) portal. The survey data was then collected online using the survey tool EvaSys. As an incentive, participants were given the option to enter a raffle for four Amazon vouchers of 50 Euros each. The recruitment mail started on May 9th, 2018. A reminder was sent out on June 22nd, 2018 and the survey was closed on August 31st.

### Italian sample

Participants were recruited *via* GenomaLab - Molecular Genetics Laboratory in Rome. GenomaLab (MGL) offers a variety of genetic testing services, including screening tests for predisposition to breast and colon cancer, cardiovascular disease, and nutrigenetic and noninvasive prenatal testing.^2^ The survey was advertised on GenomaLab’s website, and clients who had received their genetic results in the previous two weeks were invited to participate. The link to the questionnaire was sent to other clients two weeks after they had received their genetic results. Data were collected using Survey Monkey, an open source online survey application which enables users to develop and publish surveys and register responses (www.surveymonkey.com). Recruitment started in April 2018 and ended in October 2018.

## Statistical Analysis

The analysis was performed using *SPSS statistics (version 25)*. Descriptive statistics were calculated on raw data to depict the socio-demographic characteristics of both German and Italian samples. Frequencies were performed on the total distribution of our sample, whereas contingency tables and Chi-Square tests were performed to make comparisons based on country of origin, gender, age range, educational level, and parental status for each question. Expected values and residuals in every box were calculated. Contingency tables allowed us to verify whether a specific group (German vs. Italian participants) gave a significantly higher or lower rate of response (observed values) to certain items compared to the percentage expected and calculated according to the number of subjects recruited (expected values). The analysis focused on which groups agreed to certain positions and the comparison of national, gender and age differences.

## Results

A sample of 192 participants was enrolled. The response rate for Germany was 7% (n=103 of 1,517 persons contacted). Of the 1,860 Italian clients who underwent genetic testing in the period of recruitment, n = 89 completed the questionnaire, a response rate of 5%. The gender distribution reflected the overall membership distribution here.

Overall, respondents were 28% men and 69% women, with 2% not defined, and 1% not responding. 52% had previous experience with genetic testing (41% participants had no experience). The sample comprised Christians (62%), Agnostics (6%), and nonreligious people (27%). The socio-demographic characteristics of the German and Italian samples are described in **Table 1**.

**Table 1:** Sociodemographic data.

Variables	Germany		Italy	
	**N**	**%**	**N**	**%**
**Number of participants**	103	53.6	89	46.4
**Male**	41	21.4	13	6.8
**Female**	60	31.2	72	37.5
**Not defined**	2	1.0	4	1.0
**Age (years)**				
**18–25**	–	–	5	2.6
**26–35**	23	12	28	14.6
**36–50**	25	13	33	17.2
**51–70**	49	25.5	19	10
**70+**	4	2.1	–	–
**Missing**	2		4	
**Marital status**				
**Single**	21	11	15	7.8
**Married**	68	35.4	42	21.9
**Life-partnership**	7	3.6	24	12.5
**Widowed**	6	3.1	2	1.0
**Missing**	1		6	
**Number of children**				
**None**	39	20.3	35	18.2
**One**	30	15.6	21	10.9
**Two**	17	8.9	17	8.9
**Three or more**	14	7.3	2	1.0
**Missing**	3		14	
**Level of education** **Academic degree** **Vocational school** **High school** **year 10year 9** **No education**	64 7 13 14 2 0	36.3 6.9 12.7 13.7 2.0 0	45 3 28 1 3 0	24.7 3.8 35 1.3 3.8 0
**Missing**			9	

### Experience With Predictive Genetic Testing

**Figure 1:**
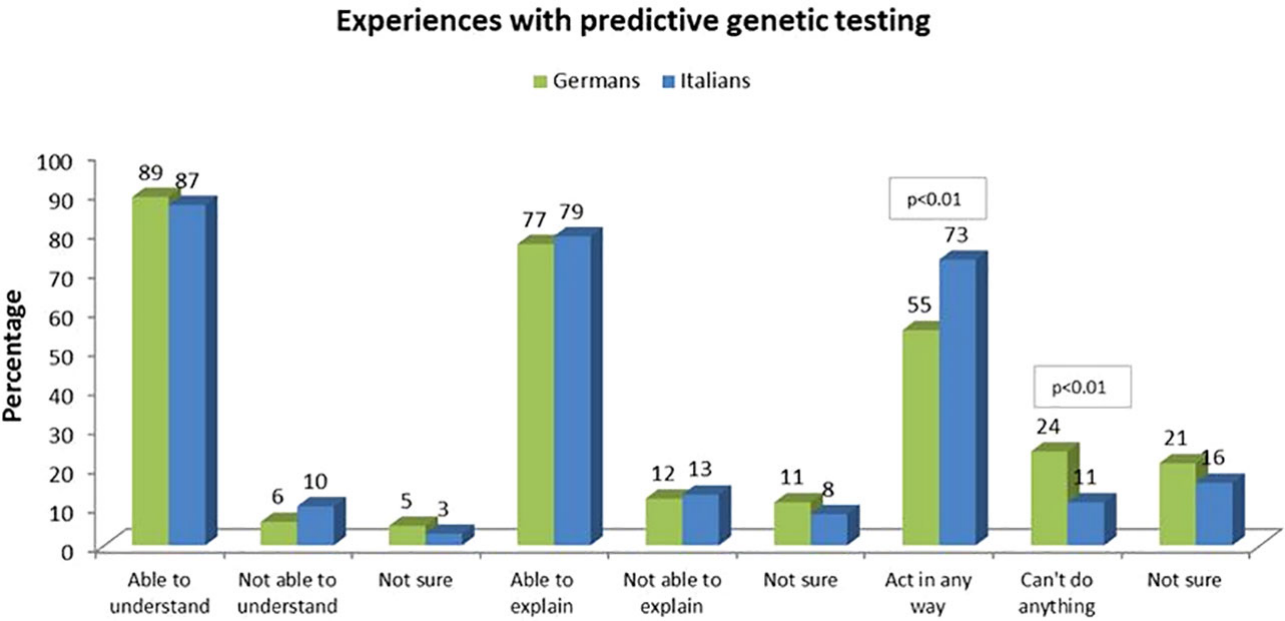
Experiences with predictive genetic testing.

When asked about their experience with genetic testing and genetic information, 89% of German participants and 87% of Italian participants answered that they understood the reports on their genetic data, while 6% of German participants and 10% of Italian participants answered that they did not understand the reports on their genetic data (**Figure 1**).

When asked if they were able to explain the results to others (e.g., family members), 77% of German participants and 79% of Italian participants answered affirmatively, while 12% of German participants and 13% of Italian participants answered negatively (**Figure 1**). No significant differences were found based on sociodemographic variables, such as gender, parental status, education, etc.

Apart from these similarities, there were significant differences among German and Italian participants: more Italian participants answered that they felt they could act in some way against a genetic predisposition (73% versus 55% of German participants), and more German participants answered that they felt they could not act in any way (24% versus 11% of Italians) (X^2^(1, N = 168) = 4.676, p < 0.01) (**Figure 1**).

### Attitudes Toward Predictive Genetic Testing

**Figure 2:**
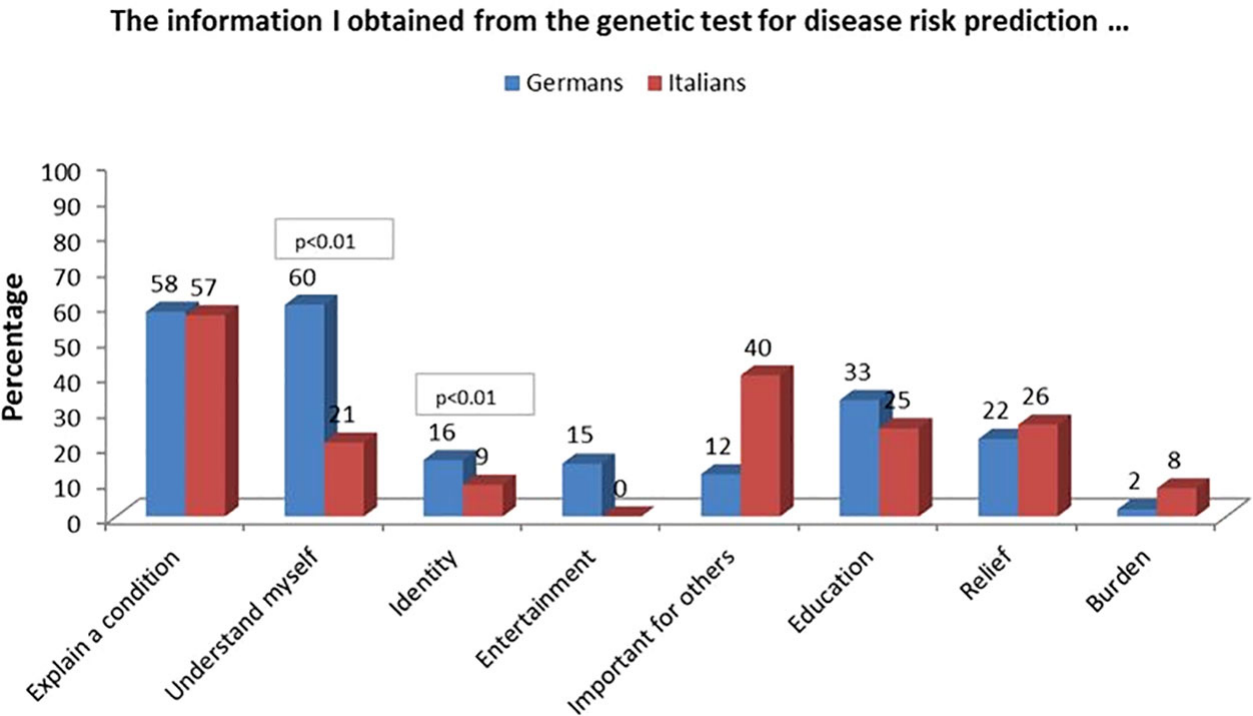
Attitudes regarding utility towards predictive genetic testing.

German participants answered more frequently that genetic testing was useful to “understand myself” (60% vs. 21% of Italian participants, with a significant difference, Χ^2^(1, N = 192) = 29.540, p < 0.01), and that genetic information had entertainment value to them (15% compared to 0% of Italians, Χ^2^(1, N = 192) = 14.060, p < 0.01). By contrast, Italian participants answered more frequently that genetic results are useful for other people, such as their family members (40% vs. 12% of German participants) (X^2^(1, N = 192 21.119, p < 0.01)(**Figure 2**).

Interestingly, only 5% of women answered that genetic information had entertainment value to them; compared to 15% of the men, with a significant difference (X^2^ (1, N = 186) = 4.676, p < 0.01).

**Figure 3:**
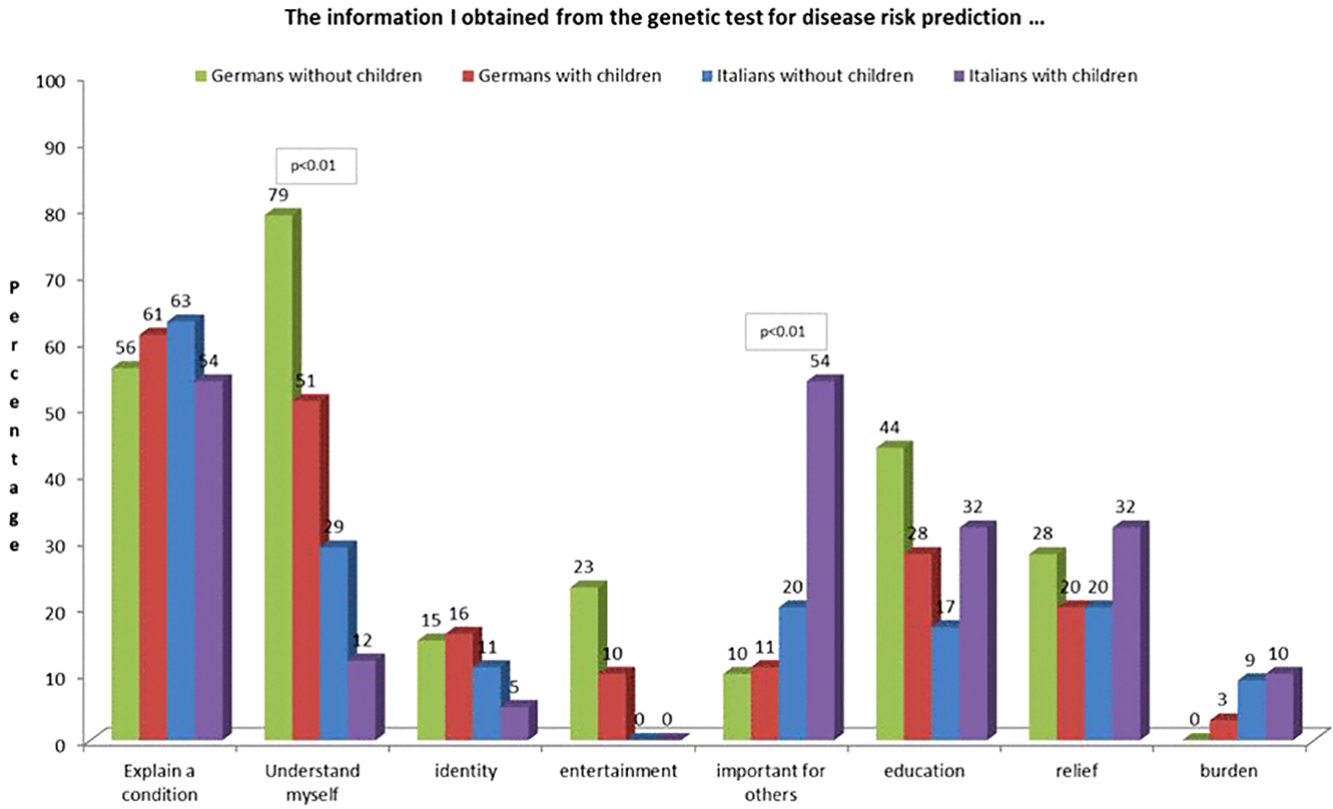
Attitudes regarding utility towards predictive genetic testing selected in with and without children.

55% of participants without children agreed that genetic testing “is helping me to understand myself” compared to 35% of participants with children, with a significant difference (X^2^(1, N = 176) = 7.049, p < 0.01). In particular, German participants without children were significantly more likely to state that genetic information “is helping me to understand myself” (79%) than Italian participants with (12%) and without (29%) children (X^2^(3, N = 176) = 41.344, p < 0.01). A similar result emerged for the question whether results have entertainment value, with 23% of German participants without children opting for this answer compared to 0% of Italian participants regardless of their parental status (X^2^(1, N = 176) = 17.812, p < 0.01). Italian participants with children considered genetic test results as “important for others (e.g., family, kids)” more frequently (54%) than German participants with (12%) and without (10%) children (X^2^(1, N = 176) = 30.335, p < 0.01) (**Figure 3**).

Across the whole sample, more participants (47%) answered that everybody should undergo genetic testing for disease risk prediction to get information about personal disease risks, against 33% of participants who were against this option, and 20% who were unsure.

**Figure 4:**
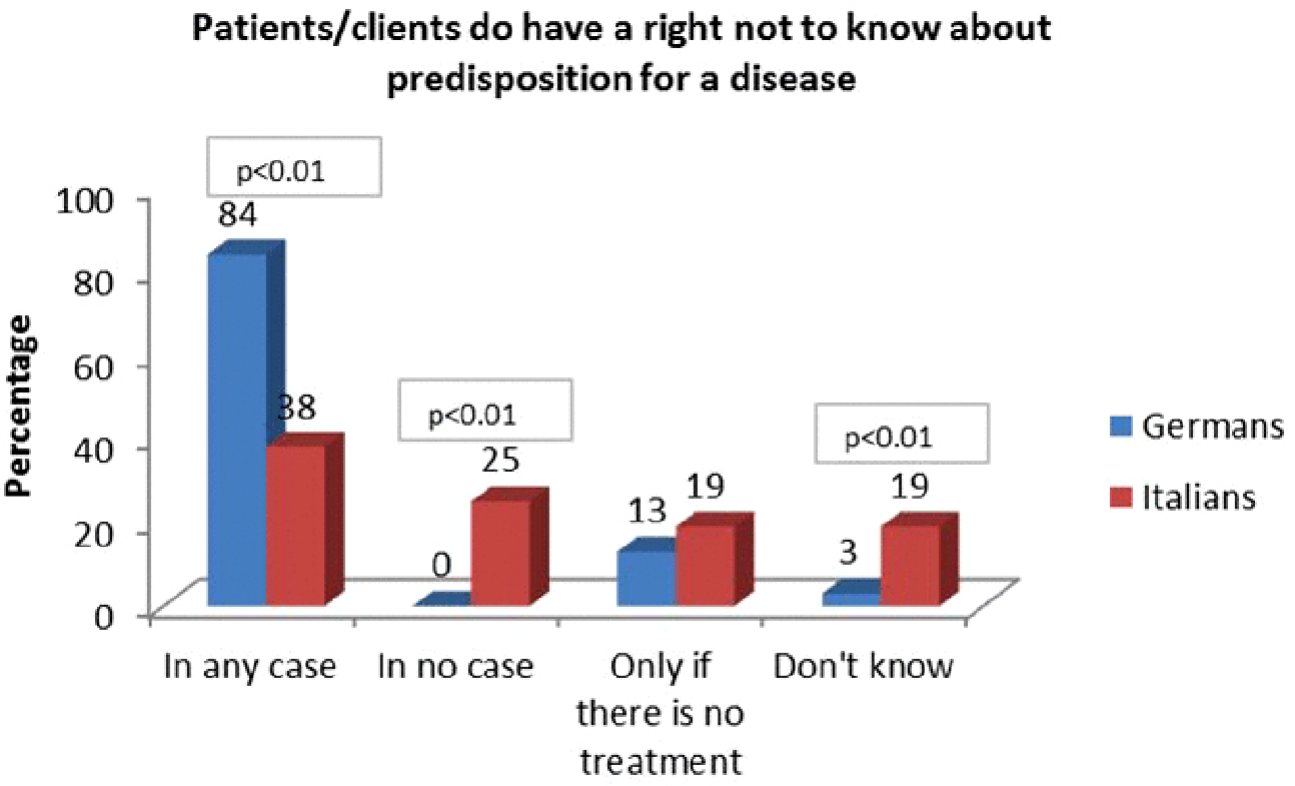
Patients/Clients do have a right not to know about predisposition for a disease.

German participants responded more often that patients/clients have a right not to know about disease predisposition regardless of the circumstances (84%), compared to Italian participants (38%), who answered more frequently that such a right exists “in no case” and “do not know”, (X^2^(3, N = 187) = 53.186, p < 0.01). Women tended to answer more frequently that patient/clients have a right not to know about disease predisposition (16%) than men (0%), this difference was significant (X^2^(3, N = 184) = 11.439, p < 0.01) (**Figure 4**).

Italian participants responding “in no case” were all women and were mostly aged between 26–35 (33%) or 36–50 (33.3%). They were predominantly married (57%) and had an academic level of education (57%). 52% had children, whereas 38% did not. 76% already had previous experience with genetic testing. They wanted to share genetic results mainly with their partner (81%), parents (67%), and children (57%), and they actually shared results with the partner (81%) and parents (76%) at roughly the same frequency as they wanted, but not with children (33%).

Interestingly, only 23% of this group of women answered that the main reasons for sharing genetic results with family members would be “the right to share”. Other responses included: 19% “have trust in others”, 10% “share the burden”, 14% “receive comfort”, 5% “feel responsible for their life”, and 38% “It is important for reproductive planning”. Most answered “They have a right to know” (47%).

**Figure 5:**
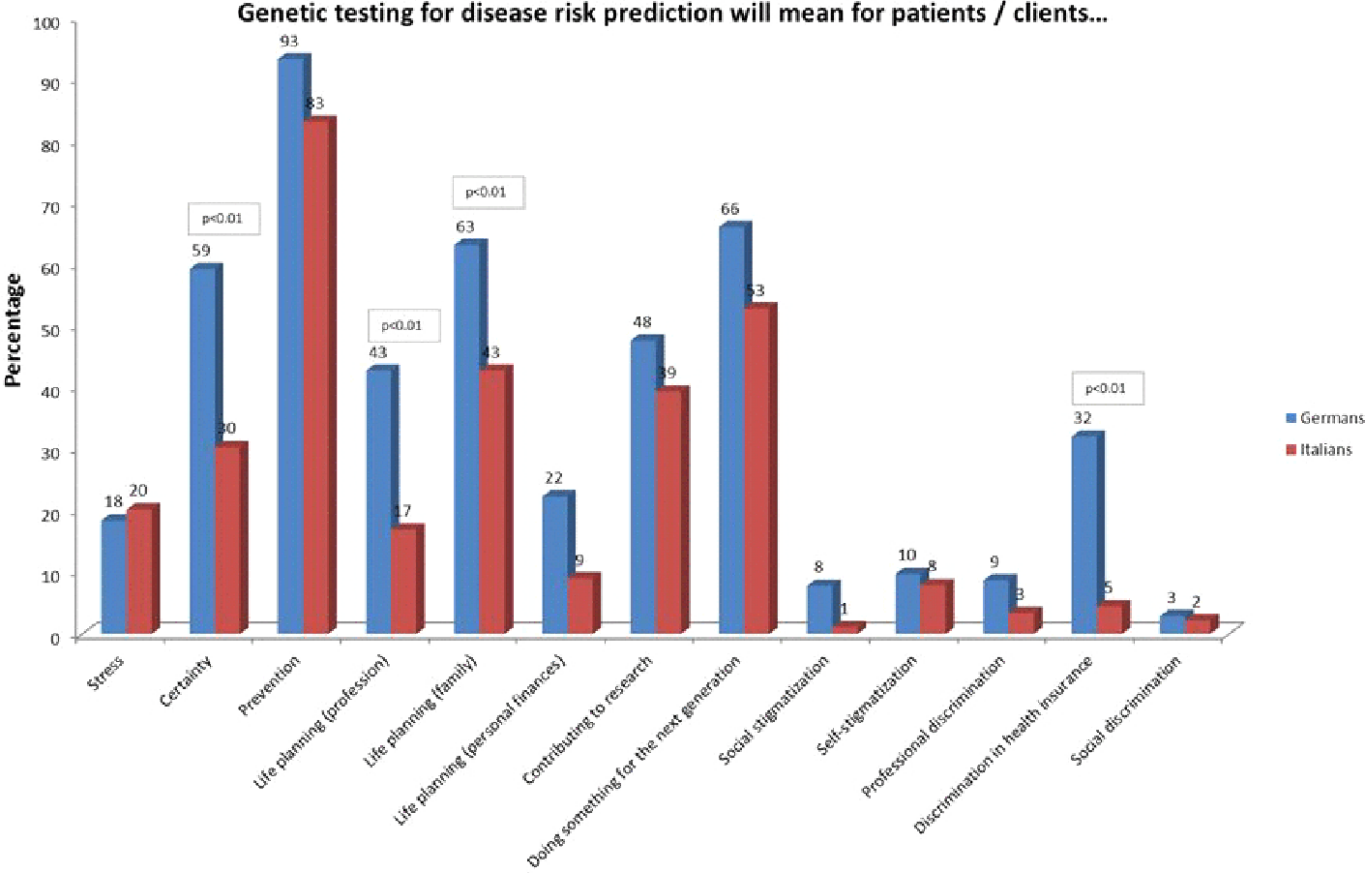
Attitudes towards opportunities and risks towards predictive genetic testing.

German participants answered more often that for them genetic information means certainty (59% vs. 30% Italians, (X^2^(1, N = 192) = 16.047, p < 0.01), and claimed that genetic testing includes preventive possibilities (93% vs. 83% Italians, Χ^2^(1, N = 192) = 4.761, p < 0.05). Significant differences were also evident regarding the perceived possibility of life planning with a view to one’s own professional life (43% German participants vs. 17% Italian participants, Χ^2^(1, N = 192) = 15.005, p < 0.01), the possibility of life planning with a view to family (63% German participants vs. 43% Italian participants, Χ^2^(1, N = 192) = 7.998, p < 0.01). German participants were also more likely to state that genetic testing involves the risk of discrimination in health insurance (32% German participants vs. 5% Italian participants, Χ^2^(1, N = 192) = 23.284, p < 0.01) (**Figure 5**).

Women declared more often than men that genetic testing for disease risk prediction means preventive possibilities (95% of women vs. 80% of men) (X^2^(1, N = 186) = 9.953, p < 0.01). More participants with a vocational school education (60%) or academic degree (39%) answered that genetic testing means a possibility of life planning with a view to one’s professional life compared to the other groups, and particularly participants with high school education (15%) (X^2^(4, N = 182) = 14,364, p < 0.01).

**Figure 6:**
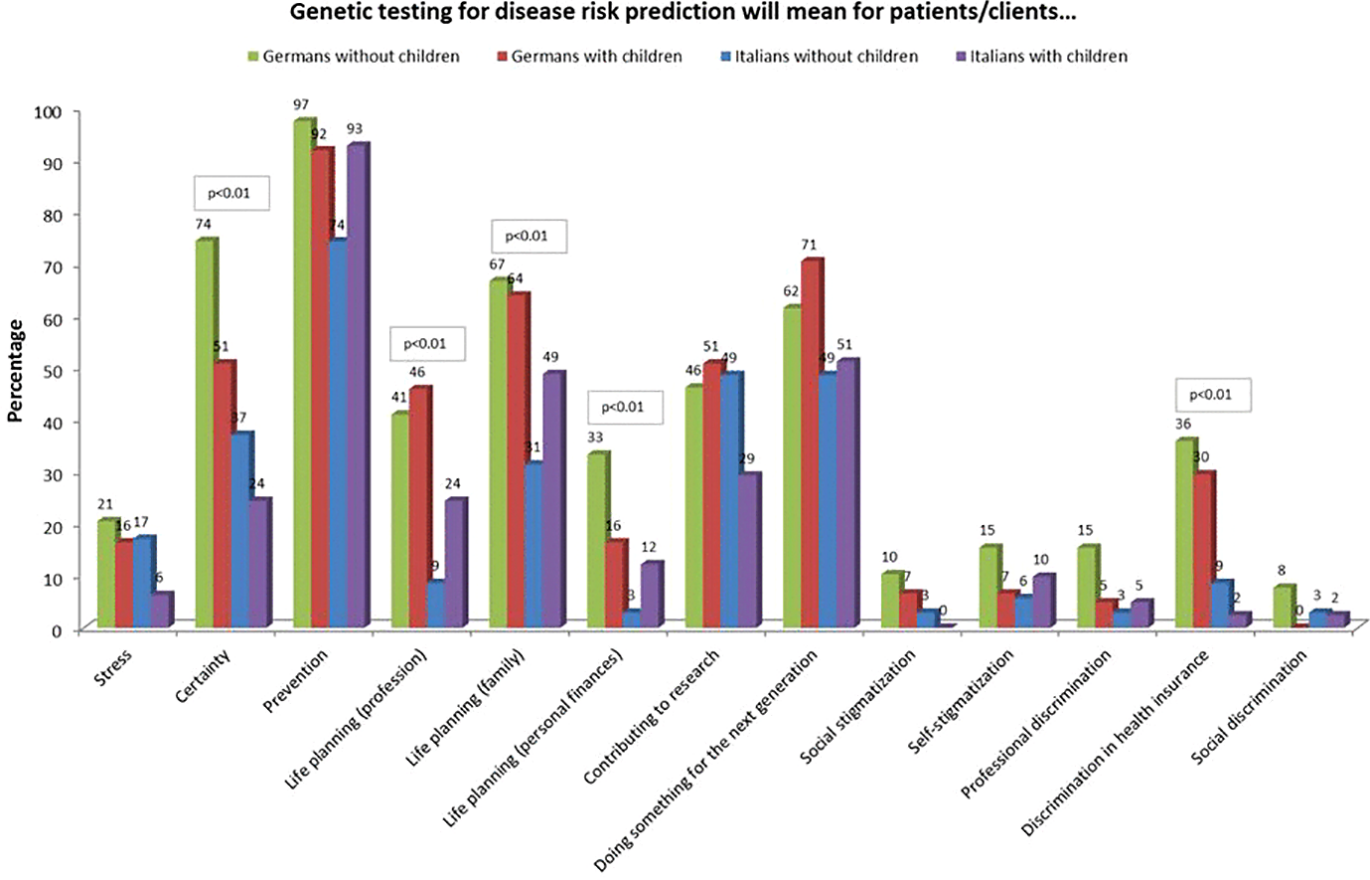
Attitudes towards opportunities and risks towards predictive genetic testing selected in with and without children.

Specifically, German participants without children answered “certainty” significantly more often (74%) compared to Italian participants without (37%) and with children (24%) (X^2^(3, N = 176) = 21.846, p < 0.01). German participants with children more often stated that genetic testing allowed the possibility of life planning with a view to profession (46%) than Italian participants without children (9%) (X^2^(3, N = 176) = 16.680, p < 0.01). Italian participants without children answered less frequently that genetic testing means the possibility of life planning with a view to family compared to the other groups (31% (X^2^(3, N = 176) = 12.573, p < 0.01). German participants without children were significantly more likely to state that there is utility in the possibility of life planning with a view to personal finances than Italians without children (45% vs. 3%, Χ^2^(3, N = 176) = 13.316, p < 0.01). German participants without (36%) and with children (30%) more often saw a risk of discrimination in health insurance than Italian participants with (2%) and without children (9%) (X^2^(3, N = 176) = 20.005, p < 0.01) (**Figure 6**).

Italian participants stated more often (74%) than German participants (47%) that predictive genetic testing is generally useful, and significantly less often that predictive genetic testing is useful in the case that an effective treatment is available (24% compared to 38% of Germans, Χ^2^(3, N = 188) = 17.557, p < 0.05). Italian participants without children answered significantly more often that genetic testing is generally useful (80%) compared to German participants with children (42%) (X^2^(4, N = 174) = 25.742, p < 0.01). German participants with children answered more frequently that genetic testing is useful in case an effective treatment is available (47%) compared to the other groups (X^2^(4, N = 174) = 25.742, p < 0.01).

When asked about regulations needed to offer genetic testing, German participants answered more frequently that genetic testing needs a standardization of test methods and limits (i.e. reliable and comparable test procedures with comparable properties) (69% vs. 51% Italian participants, Χ^2^(1, N = 192) = 6.737), medical guidelines (85% vs. 62% Italians, Χ^2^(1, N = 192) = 14.037, p < 0.01), directives for data protection (72% vs. 35%, Χ^2^(1, N = 192) = 26.396, p < 0.01), and the possibility of effective treatment (49% vs. 29% Italian participants, Χ^2^(1, N = 192) = 7.460, p < 0.01).

Men answered more frequently that a standardization of test methods and limits (76% vs. 57% women, Χ^2^(1, N = 186) = 5.961) and directives for data protection are important (70% vs. 50% women, Χ^2^(1, N = 186) = 6.451, p < 0.01). A standardization of test methods and limits was also important to participants with an academic degree (74% vs. 29% compared to the other groups. High school 42% vs. 59%; ten years of education 47% vs. 53%; < 9 years of education 40% vs. 60% Χ^2^(4, N = 182) = 17.132, p < 0.01).

### Dealing With Genetic Test Results

Italian participants preferred to involve parents more than Germans (64% vs. 42% of Germans, Χ^2^(1, N = 192) = 9.511, p < 0.01). Italian participants without children stated that they would share their test results with their parents (71%) more than German participants without children (54%) and people with children (63% Italians and 34% Germans) in general (X^2^(3, N = 176) = 15.009, p < 0.01).

More women than men stated they would share results with their parents (60% of the women vs. 37% of the men, Χ^2^(1, N = 186) = 8.010, p < 0.01). Participants with an academic degree answered (97%) they would share results with the partner more frequently than the other groups (high school diploma 83% yes; 10 years of education 67% yes, < 9 years of education 100% yes) Χ^2^(4, N = 182) = 20.407, p < 0.01).

Married participants wanted to share results with the partner (98%) more than any other group (X^2^(3, N = 178) = 24.694, p < 0.01). Significantly more participants in a life-partnership stated the intention to share results with their parents (75%) than married (44%) and single participants (64%), Χ^2^(3, N = 178) = 11.110, p < 0.01).

**Figure 7:**
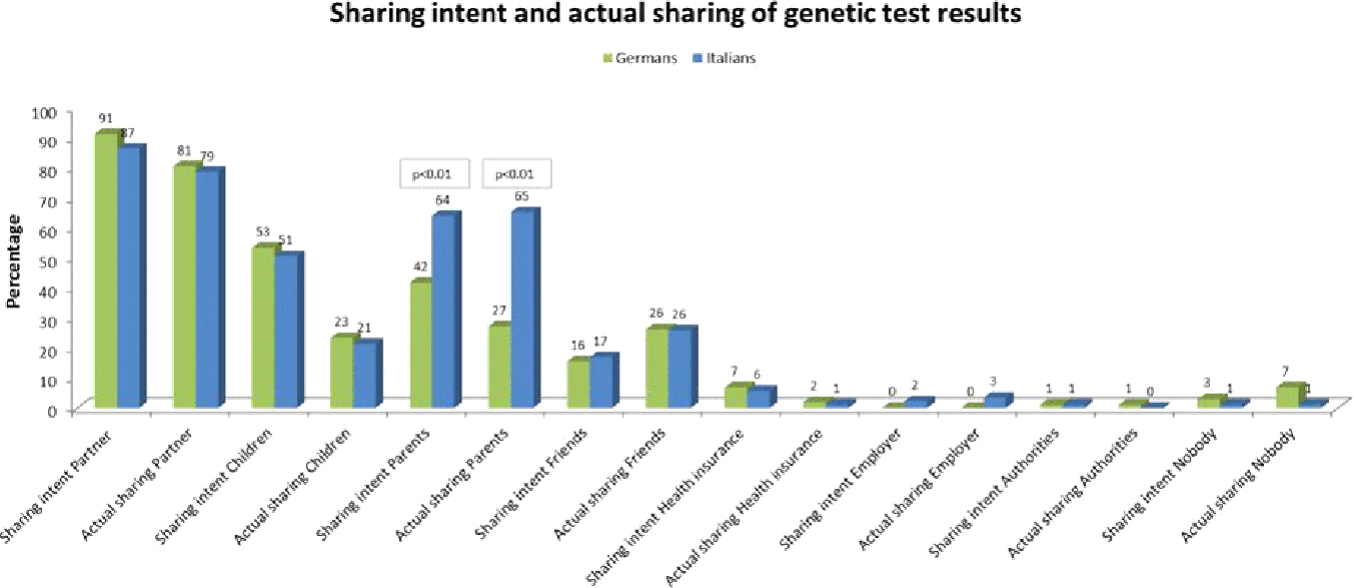
Dealing with genetic test results.

Italian participants shared results with parents more frequently than German participants (65% Italian participants vs. 27% German participants, Χ^2^(1, N = 192) = 27.857, p < 0.01) (**Figure 7**). German and Italian participants shared results with their children equally (34% German participants and 42% Italian participants). Women tended to share their results with their parents more than men (55% of the women vs. 24% of the men, Χ^2^(1, N = 186) = 15.035, p < 0.01). Overall, there was a relatively high willingness to share results within the social circle (89% with the partner, 52% with parents, 52% with their children, 16% with friends, of the whole sample of participants) while most of the participants reported reluctance to share results with employers (1%) and other institutions like health insurance (6%) (**Figure 7**).

Considering the whole sample, 80% of participants had actually shared results with the partner, 45% with the parents, 22% with their children, 26% with friends, whereas only 3 participants each actually shared results with health insurance or with the employer. Only 1 participant reported to have shared information with authorities (unspecified).

Differences were evident regarding the reasons for sharing genetic information: German participants chose the answer option “my right to test means that I can share the information” more often (54%) than Italian participants (36%) (X^2^(1, N = 192) = 6.521, p < 0.05). Similarly, German participants answered more often “I have trust in others” (44% vs. 19% of Italian participants, Χ^2^(1, N = 192) = 13.202, p < 0.01), and “I feel responsible for their [family members] life” (34% vs. 19% for Italians, Χ^2^(1, N = 192) = 5.353, p < 0.05).

German participants without children answered “…means that I can share the information” (69%, Χ^2^(1,N = 176) = 11.851, p < 0.01), and “I have trust in others” (51%, Χ^2^(1, N = 176) = 11.851) more frequently than Italian participants without children, and both German and Italian participants with children.

Italian participants wanted to share genetic results mainly with their partner (81%), parents (67%) and children (57%), and they actually shared results with the partner (81%), parents (76%) but not with children the same frequency they wanted (33.3%).

Interestingly, only 23% of Italian participants answered that the main reasons for sharing genetic results with family members would be “the right to share”, 19% “have trust in others”, 10% “share the burden”, 14% “receive comfort”, 5% “feel responsible for their life”, 38% “It is important for reproductive planning”. Most answered “They have a right to know” (47%).

Men report more trust in others than women (50% men vs. 26% women, Χ^2^(1, N = 186) = 10.219, p < 0.01), and feel more responsible for their family members’ lives (40% men vs. 22% women, Χ^2^(1, N = 186) = 6.785, p < 0.01). Participants with an academic degree answered more frequently “I have trust in others”, particularly compared to participants with high school level (39% academic degree vs. 10% high school, Χ^2^(4, N = 182) = 15.465, p < 0.01).

Men answered more frequently that “…persons or institutions can control me with the information” (9% men vs. 0% women, Χ^2^ (1,N = 186) = 12.560, p < 0.01).

## Discussion

Our results provide empirical insights to the notions of “personal utility” and “data sharing”, which are often used as umbrella terms in discussions of the usability of genomic information. The results show a relatively high willingness among participants to share information with their social circle but an overall strong reluctance to share data with official institutions (employers, health insurance) due to fear of genetic discrimination. Several studies showed that, while there are limits in regard to people’s willingness to share genetic information, there is a significant interest in sharing it for research purposes (e.g., in health data cooperatives) medical progress (Wicks et al., 2010; Haga and O'Daniel, 2011; Hafen et al., 2014; Aitken et al., 2016; Thorogood et al., 2018). This can be interpreted as openness to the shared exchange of genetic information when societal benefits are expected. The perspective may be different when it comes to sharing information with other people and institutions, such as insurance companies or employers, that have an interest other than research.

Our data also supports the idea of Wöhlke et al. (2019) that using genetic information can lead to stronger beliefs in self-efficacy. The fact that patients are willing to share their data within social groups shows that social objectives play an important role, e.g., the comparison of health data with other patients, or the exchange of information on dealing with the disease and its treatment.

The danger of stigmatization and discrimination based on genetic information is often cited as ethically problematic (DiMillio et al., 2015; German Ethics Council, 2018). However, only the German participants saw a significant danger of discrimination in health insurance, and our study showed overall little indication of fear of such negative consequences. Genetic knowledge is therefore less often perceived as a risk of individualization of health risks and loss of social solidarity, as feared by some experts (Lemke et al., 2010; Wöhlke et al., 2015). Instead, there is an apparent optimism regarding the possibilities of sharing genetic information to everyone’s benefit—a notion that also drives the development of new genetic data sharing cooperatives (see Prainsack, 2017).

It is also interesting that many participants were unsure whether it was advisable for everyone to undergo a genetic test. A clear cultural difference is evident between German respondents, who support the right not to know and find aspects of personal utility of genetic information very important, and Italian respondents, who saw the value of genetic information more in terms of one’s own and family prevention, i.e. in its potential to aid in exercising genetic responsibility (Leefmann et al., 2017). Comparing the two countries, it becomes clear that responsibility for the family was more important among the Italian respondents and that moral values are strongly influenced by this. In our view, these findings indicate a plurality of lay moralities regarding duties and rights related to genetic testing. They are in line with previous studies of affected people, which found national differences regarding the moral duty to undergo genetic testing (Raz and Schicktanz, 2009), or moral conflicts regarding whether or not one should know, and tell, in the context of Huntington’s Disease (Konrad, 2005). Our findings suggest that a possible moral obligation to share genetic information does not necessarily depend on specific conditions or predicted negative outcomes (D'Agincourt-Canning, 2001). Rather, moral obligation is closely related to family responsibility (Leefmann et al., 2017). As our results show, the Italian respondents associated a significantly higher level of family responsibility with genetic information. In contrast, German users appeared to place much more importance on individual interest and benefit.

The vast majority of our participants claimed that they understood their genetic reports. However, there are differences in the assessment of the benefits of such data: German participants were much more skeptical than Italian participants that they could counter-balance a genetic predisposition with preventive measures. In line with other empirical studies (Paton et al., 2012; Sommer et al., 2013; Lupton and Michael, 2017), we found that German participants use genetic information to learn more about themselves. In contrast, the motivation of Italian respondents in dealing with genetic information is more focused on the benefit to others, such as the family. This could be connected to the fact that in Germany there is a tendency to discuss individual genetic testing and genetic carrier screening separately (German Ethics Council, 2013). Similarly, it is striking that 1 in 4 Italians disagreed that one has a right not to know about predisposition to a disease—this right is rarely contested by experts and also exists as a legal right in both countries (German Genetic Diagnostics Act §9, (2009) Oviedo Convention 1997, Ar. 10, co 2). This could also be explained by cultural differences regarding the value of family and responsibility for others, which appears to be more significant in Italy than Germany (Rodotà, 2006). In Italy the right not to know is regulated by article 10, co. 2, of the Italian Oviedo Convention: “Everyone has the right to know all the information collected on their own health. However, the will of the person not to be informed must be respected”. Nevertheless, despite the current regulatory framework, the very nature of genetic information limits individual choice in this field, since various private law regulations affecting the family, community or society become relevant and must be adapted to the peculiar characteristics of genetic information. Therefore, with regard to genetic information, the “right for personal health” prevails (Rodotà, 2006).

Our results suggest that practices of dealing with digital health-related data vary depending on the different legal frameworks in which they are embedded. Also relevant are the respective social and cultural frameworks, which refer to standards of handling health-related data as well as the demands and acceptance of the relevant actors (Lupton, 2014). Moreover, technological progress often challenges legal frameworks with new implications. Since 2015, there is a “Law for Secure Digital Communication and Applications in Health Care” (“eHealth Law”) in Germany (German Federal digital Law, 2015). This law provides for the establishment of an electronic patient record, in which patients can store the self-collected health data and make it available to their attending physician (Federal Ministry of Health, 2015).

In addition to technical and political aspects, the resulting legal and ethical consequences must also be considered (Frizzo-Barker et al., 2016). In our view, more comparative studies on data ownership involving lay people are necessary in order to better understand cultural differences such as attitudes towards the “right not to know” in the handling of genetic digital data.

The question of benefit primarily addresses different forms of individual interest or benefit provided by genetic information that go beyond improved health outcomes, and our findings indicate that the information is used for “potential” prevention for the benefit of others (e.g., future generations, one’s own children). Cultural differences are evident in the value given to genetic information for preventing financial, family or professional problems. Those aspects were much more important to the German participants than to the Italian ones. In addition, there seems to be a cultural difference regarding the perception of genetic information as providing certainty, which was supported by about three-quarters of German participants but by far fewer Italian participants. In line with other studies, this could be an indication that in Germany genetic information is perceived to be very useful since it is a product of scientific insights and progress (Urban and Schweda, 2018).

Finally, some interesting differences emerged in our sample based on educational level and gender. It seems that people with an academic education tend to consider genetic risk information as something useful for the professional life planning and that a standardization of methods and limits for genetic analysis is paramount for them. Further, they are more interested in sharing results with their partner and have trust in other people when deciding to share their personal information such as a genetic risk predisposition. Moreover, among the Italian population, people with an academic degree also believed more in the notion that there is no “right not to know”. Our results show that people with higher education show greater openness to share this type of personal information, especially if they are generated with reliable methods, and in particular in the Italian context, excluding the right not to know”. Other studies have been conducted in the past on the attitudes toward genetic testing and their perceived utility, that have revealed differences based on the level of education, too (Haga et al., 2013; Roberts et al., 2017; Flatau et al., 2018; Schaper and Schicktanz, 2018). Our study also showed gender differences regarding the perceived utility of genetic testing and attitudes towards data sharing, such as the fact that women consider undergoing genetic testing as a preventive possibility and they want to share (and actually share) the results with parents more than men. Men on the other hand have higher privacy concerns and appear to be more interested in standardization of test methods and limits and directives for data protection, since they are worried about the possibility that persons or institutions can control them using genetic risk information. While gender differences regarding attitudes toward genetic testing have been observed in several other studies in different countries, there seems to be no clear recurring pattern this finding relates to, probably because of different studied populations and varying study designs and methods (Aro et al., 1997; Henneman et al., 2013).

## Limitations

This work is explorative in nature and subject to several limitations in regard to representativeness. Given the narrow field of research and the research question, the total target population is very small. The difference in response rates between the countries may be attributed to the use of incentives in the German setting. However, in both countries the response rate was very low, which might lead to sample bias. We cannot generalize our findings to the broader population; however, we may assume that it is somewhat representative of the smaller target population. The invitation mail in the German data collection technically allowed participants to share the link or participate in the survey multiple times. In in an unknown number of cases, doctors keep patients’ User IDs, making it impossible for the latter to respond. A limitation of the survey and related statistical analysis is the lack of continuous variables, which did not allow analysis of variance in investigating group differences.

## Conclusions

Our survey demonstrates the importance of cross-cultural comparisons (Raz and Schicktanz, 2016) to better understand national differences and similarities in lay perspectives in regard to using und sharing genetic information to indicate responsibilities and reservations. Our findings contribute to the discussion about the personal utility of genetic information. Above all, the broad spectrum of different attitudes shows that lay people see a great potential for prevention, and that predictive genetic tests will in future increase lay people’s perceived responsibility for their own health.

This raises the question of how individual autonomy and the right to know can be reconciled with the self-determination of family members and their right not to know. Predictive genetic tests can lead to an overestimation of the predictive ability of genetic information. At the same time, neglecting social risk factors for certain diseases could be both physically and psychologically detrimental for those affected. As we become increasingly exposed to genetic information in our lives, it is all the more important that we, as citizens, patients or consumers, are sensitized to, or “socialized” with, ethical questions arising from such information (Parry and Middleton, 2017; Roberts and Middleton, 2017). However, more information and educational work is needed while genetic information is combined with prevention measures aimed purely at medical interventions or family planning. In the private, family or professional spheres alike, there is a lack of information about which preventive measures can be affected by genetic knowledge. Communication challenges also arise beyond the handling of predictive information. For example, it is important not only to educate lay people about the opportunities and risks of using their genetic information, but also to avoid raising unrealistic expectations by, for example, making a factual distinction between individual therapeutic and future benefits for patients.

## Outlook

The sharing of genetic information *via* digitized patient records promises a more transparent, efficient and secure flow of information between patients, physicians and other groups in the healthcare system (Lupton, 2014). Therefore, in addition to technical and political solutions, the resulting legal and ethical consequences must also be considered (Frizzo-Barker et al., 2016). In our view, more comprehensive studies on data ownership involving lay people are necessary in order to do justice to cultural differences such as the “right not to know” in the digital age. Further, there seem to be interesting correlations between sociodemographic factors and willingness to share genetic information worth investigating. In order to evaluate future ethical problems that may arise through the integration of genetic information into eHealth and to guarantee informational self-determination, the perspectives of lay people (as users) should be taken into account along with those of experts during the development of these new digital technologies (Hartzler et al., 2013).

Since most users only partially comprehend the complex mutual relationship between data generation and use and their consequences, ethical aspects of dealing with digital health-related data, e.g. with regard to data protection and data autonomy, should be prioritized (Rothstein, 2015).

## Data Availability Statement

The datasets generated for this study are available on request to the corresponding author.

## Ethics Statement

This study was carried out in Germany in accordance with the recommendations of ‘University of Göttingen Human Research Review Committees Ref. Nr. 16/10/14’ and in Italy the research protocol was approved by the Centro Interdipartimentale di Ricerca e Intervento sui Processi Decisionali (IRIDE) and the ethical committee of the University of Milan 08/14. All subjects gave consent to participation after reviewing the study information online. The protocol was approved by the ‘University of Göttingen Human Research Review Committee’. All people enrolled in Italy gave consent to participation after reviewing the study information online.

## Author Contributions

SW, MS, and SS contributed to the design of the study, developed the survey, organized data collection in Germany and interpretation of data, and drafted the manuscript. DS (bio.logis ZfH) assisted in the design of survey and the data collection at bio.logis and worked on the manuscript. SO, IC, and FS organized data collection at GenomaLab in Italy, performed statistical analysis, including creating the figures and the table for this manuscript, and worked on the interpretation of the data. GP supported data collection and revised the contents of the *Results* and *Introduction*. All authors agreed to be accountable for all aspects of the work.

## Funding

This research is developed within the framework of Mind the Risk [funded by the Swedish Riksbankens Jubileumsfond, 2015–2019 (grant no. 1351730)].

## Conflict of Interest

Author DS is founder of the company bio.logis genetic information management.

The remaining authors declare that the research was conducted in the absence of any commercial or financial relationships that could be construed as a potential conflict of interest.

## Footnotes

1 A guest login to the genetic information services is available via https://my.pgsbox.de, username: SurveyGER-I, password: GeneticInformation2019.

2 https://www.laboratoriogenoma.eu – a guest login to the services is available via https://www.genomagroup.com/LoginRefertazione.aspx?ln=EN, username: SurveyITA-G, password: GeneticInformation2019a! (Access 25. September 2019).
