# Supplementary material for: German and Italian Users of Web-Accessed Genetic Data: Attitudes on Personal Utility and Personal Sharing Preferences. Results of a Comparative Survey (n=192)
Source: Front Genet. 2020 Mar 18;11:102. doi: 10.3389/fgene.2020.00102 (PMC7099127; doi:10.3389/fgene.2020.00102)
Supplement: Supplementary file 1 [file Table_1.doc]

# Supplementary Material 1

# Survey draft “Attitudes about handling genetic information”

Conceptualized by: Sabine Wöhlke, Manuel Schaper, Silke Schicktanz

Update: 23 Mar 2018

Dear Participant,

Thank you for taking time to participate in our survey. With your participation you will support a scientific study by …………………………(please add).

Filling out the survey takes app. 10 minutes. Unless specified otherwise, please check one box per question. If you are not sure what to answer or wish not to answer a question, you can skip it.

To thank your gesture, we raffle four amazon vouchers of 50 Euro each among all participants.

If you wish to take part in the raffle, please enter you e-mail address at the end of the survey, so we can contact you. For this purpose, you will be redirected to a web page of …………………… (please add).

Thanks for your support.

…………………………..

**1. Ihre**

# I –Experience with Genetic Testing

1. Were you satisfied with the genetic test results?

O Yes

O No

O Not sure

1. Were you able to understand the genetic test results?

O Yes

O No

O Not sure

1. Are you able to explain the meaning of the genetic test results to others?

O Yes

O No

O Not sure

1. If you have undergone testing for predisposition to a disease: Do you feel you can do anything about how the predisposition affects you and your family?

O Yes

O No

O Not sure

# II – Questions about your opinion on genetic testing

1. Please check all boxes that apply to you: “The information I obtained from the genetic test for disease risk prediction…” (multiple answers possible)

O …is helping to explain a condition I have

O …is helping me to understand myself

O …is relevant for my identity

O …is entertaining me

O …is important for others (e.g. family, kids)

O …is educating me

O …provides relief

O …is a burden

1. Everybody should undergo genetic testing for disease risk prediction to get information about personal disease risks.

O Yes

O No

O Not sure

1. Patients / clients do have a right not to know about predisposition for a disease…

O In any case

O In no case

O Only if there is treatment

O Don’t know

1. In my opinion, genetic testing for disease risk prediction will mean for patients / clients… (multiple answers possible)

O High psychological stress

O Certainty

O Preventive possibilities

O Possibility of life planning with a view to profession

O Possibility of life planning with a view to family

O Possibility of life planning with a view to personal finances

O Possibility of contributing to research

O Possibility of doing something for the next generation / own kids

O Risk of social stigmatization/social depreciation

O Risk of self-stigmatization/self-depreciation

O Risk of familiar discrimination

O Risk of professional discrimination

O Risk of discrimination in health insurance

O Risk of social discrimination

O Other_______________________

1. Imagine there is a genetic test to predict the risk of getting a disorder (so-called predictive genetic tests) for a variety of future diseases. There aren’t any unintended side effects, it is easy to complete and is covered by national health insurance companies. All women and men between 40 and 50 are tested regularly to assess their disease risks.

What is your opinion on such a possibility?

O I believe that predictive examinations are generally useful

O I believe that predictive examinations are useful in case an efficient treatment is available

O I believe that predictive examinations are not useful

O I believe that predictive examinations are only of limited use because_____________________________________________

1. In my opinion, the following regulations are necessary to offer genetic testing for predisposition to a disease: (multiple answers possible)

O Standardization of test methods and limits

O Medical guidelines on patient’s information and on dealing with the results

O Directives for data protection

O Possibility of efficient treatment

O Coverage by health insurance

O No further directives are necessary

O Other_____________________________________________

# III – Questions about handling test results

1. Which of the following would you definitely share your test results with: (multiple answers possible)

O My Partner
O My Children
O My Parents
O My Friends
O My Health Insurance
O My Employer
O Authorities
O Nobody
O Other _____________________________________________

1. With whom have you shared your genetic test results so far? (multiple answers possible)

O My Partner
O My Children
O My Parents
O My Friends
O My Health Insurance
O My Employer
O Authorities
O Nobody
O Other _____________________________________________

1. In your opinion, what would be the main reasons for sharing your genetic results with family members? (multiple answers possible)

O My right to test means that I can share the information

O I have trust in others

O I want to share the burden

O I want to receive comfort / understanding for my condition

O I feel responsible for their life

O It is important for reproductive planning

O My family members have a right to know
O Other __________________________________

1. If you have not shared your results with anyone in particular, what are your moral reasons for this? (multiple answers possible)

O I have the right not to tell

O The respective persons or institutions have a right not to know

O The respective persons or institutions might misuse the information

O The respective persons or institutions can control me with the information
O Other _____________________________________

# IV – Questions about you

1. Age

O 18-25

O 26-35

O 36-50

O 51-70

O 70+

1. Gender

O Male

O Female

O Neither
O Rather not say

1. Highest degree

O 9 years

O 10 years

O High School

O Vocational School

O Academic Degree

O None / Rather not say

1. Do you have previous experience with genetic testing?

O Yes

O No

1. Religion

O Christianity

O Islam

O Judaism

O Buddhism

O Hinduism

O Agnostic

O None

O Other _____________________________________________

1. Marital Status

O Single

O Married

O Life-partnership

O Widowed

1. Number of kids

O None

O 1

O 2

O 3 or more

1. Average time spent online per day

O 0 – 1 hours

O 1 – 2 hours

O 2 – 4 hours

O 4 – 6 hours

O 6 hours or more

O None

1. Thank you for answering our questions! If you would like to share anything or comment on the questionnaire, please use this field!

If you want to take part in the raffle, please leave your contact data here. An e-mail address will do.
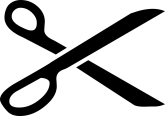
The contact information will be stored and processed separately. For this purpose, you will be redirected to another website so it is not possible to trace back a single person.
